# Virtual Reality Is an Effective Tool for Learning Techniques in Arthroplasty: A Systematic Review and Meta-Analysis

**DOI:** 10.5435/JAAOSGlobal-D-23-00078

**Published:** 2023-06-19

**Authors:** Nicholas J. Pettinelli, Amy Y. Lee, Michael S. Lee, Ronak J. Mahatme, Stephen M. Gillinov, Andrew E. Jimenez

**Affiliations:** From the College of Osteopathic Medicine, Kansas City University, Kansas City, MO (Pettinelli); the Medical College of Wisconsin, Milwaukee, WI (Lee and Lee); the University of Connecticut School of Medicine, Farmington, CT (Mahatme); and the Department of Orthopaedics and Rehabilitation, Yale School of Medicine, New Haven, CT (Gillinov and Dr. Jimenez).

## Abstract

There is a scarcity of aggregate literature reporting on the efficacy of virtual reality (VR) concerning surgical skill development within the realm of arthroplasty. The purpose of this study was to evaluate the effect of VR on the training of orthopaedic surgery residents and medical students learning surgical techniques in arthroplasty.

**Methods:**

A systematic review was conducted querying PubMed, Cochrane Trials, and Embase in September 2022. Studies were included if they involved education or training of orthopaedic surgery residents/students, used VR, and reported on outcomes measuring surgical skills related to arthroplasty. Outcomes present in a minimum of three studies underwent additional statistical analysis with forest plots.

**Results:**

Seven studies met inclusion criteria and included a total sample size of 148 residents/students between MS4-PGY6. Five of the seven included studies showed VR to be an effective training modality, with two studies demonstrating that VR reduced the error rate (*P* < 0.05). The two most reported outcomes were procedure duration and objective structured assessment of technical skills. Orthopaedic trainees using VR conducted procedures in markedly less time than control groups (SMD, −0.81 minute; 95% confidence interval, [−1.45 to −0.17 minutes]; *P* = 0.01). No significant difference was found comparing objective structured assessment of technical skills between VR and control groups (SMD, 0.44; 95% confidence interval, [−1.05 to 1.93]; *P* = 0.56).

**Conclusion:**

Although the extent to which VR can outright replace standard learning modalities is unclear at this time, its usefulness as a supplemental learning modality in arthroplasty, especially in the absence of available on-demand resources, may be of value. A paucity of literature exists to evaluate the effect of a longitudinal VR curriculum on direct patient care performance by orthopaedic surgery residents learning techniques in arthroplasty, necessitating additional study.

Virtual reality (VR) is an evolving technology that seeks to recreate and simulate an interactive environment often with a head-mounted display. VR is “immersive” in the sense that it provides a 3D environment that allows for 360° immersion with the ability of users to manipulate virtual items through handheld devices.^1^ VR has been applied to a multitude of surgical fields that have reported improvement in trainee performance on tasks ranging from cochlear implantation,^2^ cataract surgery,^3^ and laparoscopic surgery.^4^

There are several advantages to the use of VR in surgical training, including the fact that VR offers an opportunity for surgical trainees to learn and practice in a safe environment with minimal risk to patients,^5,6^ a quality which has been demonstrated to improve skills in the OR.^7^ Furthermore, VR offers the opportunity to assess trainees in a consistent and reproducible manner that does not depend on the availability of cases they encounter at their home institution or in cadaveric specimens.^8,9^ With a combination of head-mounted displays and handheld devices capable of providing haptic and physical feedback, VR can track hand movements with incredible accuracy.^10^ Integral to this movement tracking is the recording of performance metrics, specifically those that allow learners to pinpoint areas which require improvement.^11^ When compared with standard training, VR enhances surgical performance, decreases procedure times, and reduces the amount of intraoperative error.^12^

Of note is that VR can be used as a training tool specifically within the realm of orthopaedic surgery. Recent studies seem to demonstrate that orthopaedic surgical training with VR is of either equivalent or superior efficacy to traditional training modalities.^8^ This has led to the investigation of VR usage within the training of orthopaedic surgery residents, where recent studies have suggested that training with VR can improve surgical performance.^13,14^

Regarding VR training within orthopedics, most current research has shown success in developing surgical skills related to arthroscopy.^15-18^ Furthermore, recent analysis of the literature has concluded that VR training is an effective tool to develop skill and technique within orthopaedic surgery as a whole.^8^ With the advancement of VR technology, the scope of procedures that are available through VR training has expanded to include other areas in orthopaedics, such as arthroplasty.^19^

Arthroplasty presents some inherent challenges to learning that can be difficult to overcome as a surgical trainee, such as learning the instrumentation, array of appropriate implants, movement efficiency, and attention to detail.^20^ Unicompartmental knee arthroplasty, for example, is a nuanced procedure requiring intimate familiarity with each of its many components and steps.^21^ For many surgical residents, it can be difficult to access interactive training modalities such as sawbone or cadaver preparations equipped with arthroplasty-specific instrumentation on an as-needed basis. Thus, many surgical trainees are limited to preparation with more passive modalities, such as reading a technique guide or watching an instructional video. To help ameliorate this issue, VR has been suggested as a potential adjunct to surgical education that provides surgical residents with the convenience of active learning on demand.

Currently, there is a paucity of aggregate literature examining the efficacy of VR as a training tool for the development of surgical skills specific to arthroplasty. The purpose of this systematic review and meta-analysis was to evaluate the effect of VR on the training of orthopaedic surgery residents and medical students learning surgical techniques in arthroplasty. It was hypothesized that VR training would demonstrate notable improvement in the development of arthroplasty-related surgical skills.

## Methods

### Study Search and Identification

A systematic review of the literature was conducted with the following keywords: (virtual OR augmented) AND (reality OR simulation) AND (arthroplasty) in PubMed, Embase, and the Cochrane Library (Table 1) in September 2022 using the preferred reporting items for systematic reviews and meta-analyses (PRISMA) guidelines.^22^ Two authors, (X.X.) and (Y.Y.) used the Covidence screening tool (Covidence systematic review software, Melbourne, Australia, 2022) to screen titles/abstracts and review full texts in the search and met a consensus for all articles with inclusion potential. Studies were included if they involved education or training of orthopaedic surgery residents/students, used VR, and reported on outcomes measuring surgical performance related to arthroplasty as presented in Supplemental Table 2 (http://links.lww.com/JG9/A288). If reviewers did not agree after initial evaluation, articles underwent re-review with discrepancies discussed with the third author (Z.Z.) until an agreement on inclusion was made. Articles defined as case reports, technical notes, opinions, abstracts, book chapters, and systematic reviews/meta-analysis as well as those not written in English were excluded from this study.

**Table 1:** Databases Used and Relevant Search Strategy

Database	Search Strategy	Items Found
PubMed	(virtual [title/abstract] OR augmented [title/abstract]) AND (reality [title/abstract] OR simulation[title/abstract]) AND (arthroplasty[title/abstract])	120
Cochrane	#1 (virtual) ti, ab, kw OR (augmented) ti, ab, kw	63
	#2 (reality) ti, ab, kw OR (simulation) ti, ab, kw	
	#3 (arthroplasty) ti, ab, kw	
	#4 (AND #1-#3)	
	#4 used for article search in cochrane	
Embase	(“VR”: ti, ab OR “augmented reality”: ti, ab OR “simulation”: ti, ab) AND (arthroplasty: ti, ab)	147

ab = abstract, kw = keyword, ti = title, VR = virtual reality.

### Quality Assessment

The Cochrane risk of bias tool was used to grade six articles in this study, and the methodologic index for nonrandomized studies criteria was used to grade one article included in the study that did not randomize patients.^23,24^ The Medical Education Research Study Quality Instrument (MERSQI) was also used to measure the study quality of all included studies.^25^ Two authors (X.X. and Y.Y.) scored all articles until consensus was met. If articles had initial different scores, they underwent re-review until an agreement was made. All levels of evidence were established using the criteria set forth by Hohmann et al.^26^

### Data Extraction

Title, author, publication date, study design, study period, demographics of intervention and control groups (number of trainees, surgical training level, and age), VR training intervention, control training, surgical procedures, measures of surgical skill and competency, including objective structured assessment of technical skills (OSATS) and procedure-based assessment and associated *P*-values, and procedure durations for both intervention and control groups were recorded. All extracted data were recorded in Google Sheets (Mountain View, CA).

### Statistical Analysis

Descriptive statistics (means, percentages, and standard deviations) are presented where applicable. A meta-analysis was conducted, and forest plots were created for outcomes present in three or more studies, such as OSATS and procedure duration. Heterogeneity was assessed using the I^2^ statistic, and significance was defined as *P* < 0.05. Heterogeneity was considered low with any I^2^ values below 40%, moderate at values above 40%, and high at values greater than 75% based on previously established cutoffs.^27^ These outcomes were compared using Cochrane Reviewer Manager web application (computer program, version 5.4, the Cochrane Collaboration, 2020).

### Prospective Reporting

This study evaluated published outcomes concerning medical student and orthopaedic surgery resident surgical performance and, therefore, did not require institutional review board approval. The data outcomes analyzed and reported here consisted of data extracted from published primary literature measuring outcomes of medical student/orthopaedic surgery resident surgical performance rather than patient outcomes. As such, the authors of this article did not pursue prospective registration/reporting to the PROSPERO International Prospective Register of Systematic Reviews.

## Results

### Search Results

**Figure 1:**
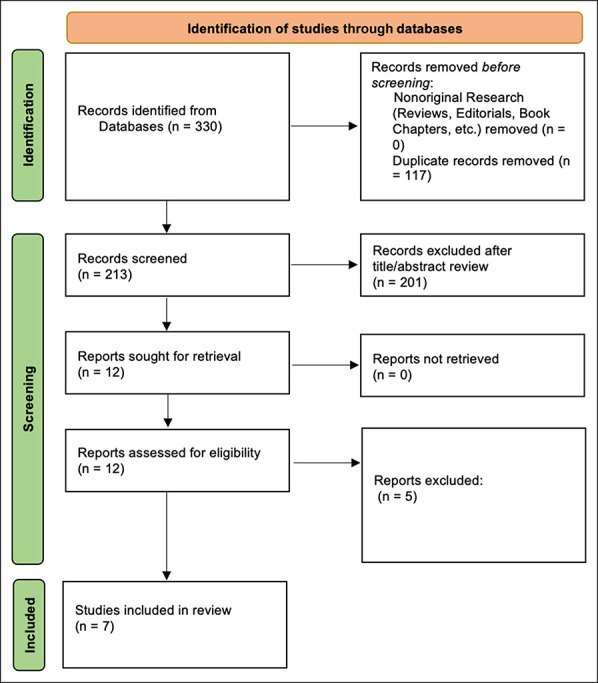
PRISMA flowchart showing studies identified for inclusion in review. PRISMA = preferred reporting items for systematic reviews and meta-analyses.

The systematic search yielded a total of 330 studies for screening, with 213 studies remaining after the removal of 117 duplicates. Twelve studies then underwent full-text review and were assessed for eligibility using the inclusion and exclusion criteria presented in Table 2. After this process, seven studies met the criteria for this study (Figure 1). Six of the included studies were level II,^19-21,28-30^ and one study was level III.^31^

**Table 2:** Inclusion and Exclusion Criteria for Full-Text Review of Relevant Primary Research Articles

Inclusion Criteria	Exclusion Criteria
Orthopaedic surgery residents, fellows, medical students	Wrong population: scrub techs/nurses
Used VR	Augmented reality, mobile applications
Reported outcomes related to surgical performance in arthroplasty	Case reports, technical notes, opinions, abstracts, book chapters, and systematic reviews/meta-analysis
Written in English	

VR = virtual reality.

### Demographics

Descriptive information of the included studies regarding study type, MERSQI score, surgical trainee level of education, arthroplasty procedure, and intervention control are listed in Table 3. In this study, 148 surgical trainees with varying levels of education from MS4 to PGY6 were included. Hooper et al^28^ analyzed post-VR performance exclusively in first-year orthopaedic surgery residents. Zaid et al^21^ included the greatest range of trainee surgical education, from fourth year medical students to orthopaedic surgery fellows. Six studies sought to compare the performance of orthopaedic surgical trainees randomly designated to either a VR training or control training group.^19-21,28-30^ One study by Logishetty et al^31^ assessed improvements in surgical skills after all patients received training with VR and was not randomized.

**Table 3:** Demographics of Included Primary Literature

Author and Year	LOE	Study Type	Intervention Period	MERSQI	Trainee Sample	Intervention Control	Cadaver-Based Procedure
Logishetty et al^31^ 2020	III	Before/aftert the cohort study	6 wk	11.5	32 PGY1-4	Baseline test	AA THR
Zaid et al^21^ 2022	II	RCT	45 min of VR	15.5	22 MS4-PGY6 (11:11)	Traditional technique guide	UKA
Logishetty et al^19^ 2019	II	RCT	6 wk	15.5	24 PGY3-5 (12:12)	Conventional training	AA-THA
Hooper et al^28^ 2019	II	RCT	2 wk (two VR trials)	13.5	14 PGY-1 (7:7)	Standard study materials	THA
McKinney et al^20^ 2022	II	RCT	1 hr	15.5	22 PGY1-5 (11:11)	Traditional technique guide	UKA
Lohre et al^29^ 2020 (glenoid exposure)	II	RCT	One session	17.5	16 PGY4/5 (8:8)	Technique article	Glenoid exposure
Lohre et al^30^ 2020(reverse shoulder)	II	RCT	One session	17.5	18 PGY4/5 (9:9)	Video training	RSA

AA = anterior approach, LOE = level of evidence, MERSQI = medical education research study quality instrument, PGY = postgraduate year, RCT = randomized control trial, RSA = reverse shoulder arthroplasty, THA = total hip arthroplasty, THR = total hip replacement, UKA = unicompartmental knee arthroplasty.

**Figure 2:**
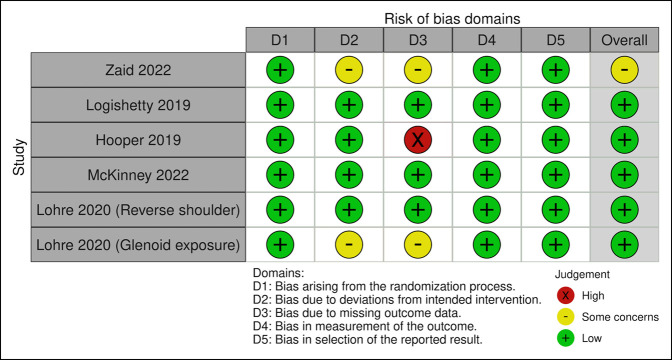
Illustration showing Cochrane risk of bias assessment for randomized trials (Risk of Bias 2.0) for included studies.

All seven of the included studies achieved a MERSQI score of at least 11.5^19-21,28-31^ of 18, where a mean score of 10.7 is generally considered an acceptable cutoff value for methodological quality in medical education research.^32^ One of the included studies raised some concerns in the overall Cochrane risk of bias assessment Figure 2.^21,33^ The one included study with a nonrandomized study population received a 20 methodological index for nonrandomized studies criteria grading.^31^

The seven included studies varied in the cadaver-based open procedures on which surgical trainees were assessed and included total hip arthroplasty,^19,28,31^ unicompartmental knee arthroplasty,^20,21^ reverse shoulder arthroplasty,^30^ and glenoid exposure task.^29^ The most prevalent procedure assessed was total hip arthroplasty in three of the seven included studies^19,28,31^ (Table 3).

### Outcomes of Surgical Performance

**Figure 3:**
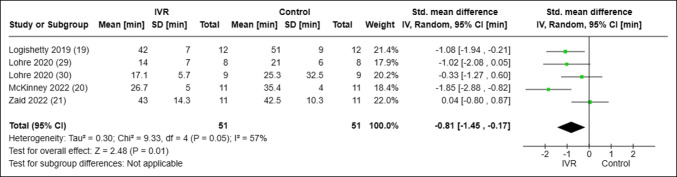
Forest plot analyzing the procedure duration in trainees using VR versus control learning modalities. CI = confidence interval, IV = intravenous, IVR = immersive virtual reality, SD = standard deviation, VR = virtual reality.

**Figure 4:**
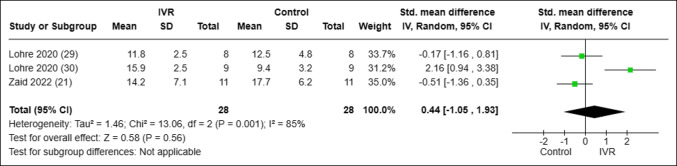
Forest plot analyzing the mean OSATS scores in trainees using VR versus control learning modalities. CI = confidence interval, IVR = immersive virtual reality, OSATS = objective structures assessment of technical skills, VR = virtual reality.

All seven of the included studies reported on some form of objective surgical skill assessment as presented in Supplemental Table 1 (http://links.lww.com/JG9/A287). Six of the included studies reported on procedure duration times.^19-21,29-31^ However, Logishetty et al^31^ compared metrics in the same group of nonrandomized trainees before and after VR training and thus were excluded from pooled statistical analysis. When comparing procedure duration across studies, orthopaedic surgery trainees learning through VR demonstrated markedly faster completion of their respective procedures compared with their counterparts learning through control modalities (standardized mean difference of −0.81 minutes; 95% confidence interval [CI], [−1.45 to −0.17 minutes], I^2^ = 57%, *P* = 0.01) Figure 3. Three of seven studies used and reported on the OSATS.^21,29,30^ When analyzed across studies, no notable difference was found when comparing OSATS in VR versus control training (standardized mean difference of 0.44; 95% CI, [−1.05 to 1.93], I^2^ = 85%, *P* = 0.56) Figure 4.

## Discussion

The main findings of this study were that (1) medical students and resident surgeons learning arthroplasty techniques through VR conducted respective procedures in less time than their non-VR counterparts (Standardized Mean Difference [SMD], −0.81 minute; 95% CI [−1.45 to −0.17 minutes], I^2^ = 57%, *P* = 0.01) and 2) mean OSATS scores demonstrated no statistically significant difference between the VR-trained group and control group (SMD, 0.44; 95% CI [−1.05 to 1.93], I^2^ = 85%, *P* = 0.56).

It is possible that training with VR facilitates greater engagement of surgical trainees with the surgical technique. Because VR necessitates the active participation of trainees and practiced rehearsal of the steps required to complete surgery, it stands to reason that VR-trained surgical trainees would complete assigned tasks more quickly as demonstrated in the procedure duration subanalysis of this study. In comparison, traditional learning modalities, such as instructional videos or surgical technique articles, constitute passive learning with less engagement of the material by surgical trainees. Active learning has been associated with an increased transfer of learned concepts to clinical competence and provides increased motivation for self-directed learning.^34^ Furthermore, improved procedure time can be beneficial in the clinical setting. Within knee arthroplasty, increased procedure durations have been linked to increased infection rates and increased duration of hospital stays as well as costs.^20,35^ A recent systematic review of immersive VR across several different surgical subspecialties similarly demonstrated consistently shorter time to completion in VR-trained groups.^9^

There was no notable difference in the OSATS score between VR-trained group and control group performance in the three included studies that used this tool. Although various factors may contribute to the lack of notable difference in the OSATS scores, this result went against findings from multiple studies that used an internally developed rating scale that showed a markedly decreased error rate, decreased procedure time, and increased task specific checklist scores for VR-trained groups.^9,19,20,34^ It should also be noted that the I^2^ statistic corresponded to a higher level of caution interpreting the results because of heterogeneity.

Although OSATS stand out as a validated objective measure of orthopaedic procedures incorporating procedure time, instrument handling, flow of operation, and knowledge of procedure, numerous other factors should be accounted for such as implant position and/or encountering adverse events.^1^ OSATS also may not be the most effective tool in measuring quality of surgical results because OSATS scoring depends on external evaluation of clinical competencies rather than other key elements of surgical competencies such as surgical outcomes, postoperative complications, and necessity for revisional surgery.^36^

An issue preventing progress in this arena is the lack of a standardized measurement tool that allows pooled analysis. However, the ability of VR software itself to track and analyze this information is showing an upward trajectory in its ability to assess users within multifactorial parameters. One such metric, the PrecisionScore serves as a composite of multiple metrics and has been validated by Lohre and colleagues.^30^ A single, comprehensive measurement tool such as this one could not only aid residency programs in assessing trainee progress toward surgical competency^1^ but also help improve the ability of researchers to analyze VR's effect on training surgeons more effectively.

Interestingly, the resources available to train orthopaedic surgery residents can vary markedly from one program to another. In a survey study completed in 2015, it was found that only 9.8% of residency programs used VR as a training resource for arthroscopic surgery.^37^ It can be estimated that the number of residency programs offering VR as a training resource has increased since the time of this study, but presently this scarcity may offer some insight into the lack of multicenter studies regarding VR efficacy.

Opponents to VR within residency programs might be likely to cite costs as an obstacle barring its implementation. However, instruments such as head-mounted displays cost as little as $400, with the brunt of a $4000 to 8000 price tag attributed to software licenses and usage.^1,28^ One study has even estimated VR to be 34.1 times more cost-effective than traditional training methods.^30^ An additional factor supporting the usage of this technology in medical education is that it has been regarded positively by participants using VR. A systematic review and analysis of 41 studies spanning a wide application of VR to medical education found most participants preferring VR over traditional training tools.^38^

Only recently, a case report was able to identify a scenario where VR training was used just before direct patient care in the OR, highlighting the potential for this modality to prepare orthopaedic surgery residents for infrequently encountered cases.^39^

As presented in Table 3, the studies assessing VR in arthroplasty primarily have assessed trainees after one or very few training sessions. Although an extreme example by Walbron et al^18^ demonstrated improved instrument handling and procedure times compared with control subjects when PGY-1 trainees received at least 10 hours of independent practice in a 6-month period on a VR arthroscopy (non–head-mounted) simulator. As a result, more studies evaluating surgical trainee performance from longer term sustained that practice with VR could improve our understanding of its efficacy in surgical training.

### Strengths

This study has several strengths. It is the first study to identify and aggregate existing literature analyzing VR efficacy pertaining specifically to arthroplasty. Furthermore, the population in this study examines a wide range of medical trainees, from fourth-year medical students to orthopaedic surgery residents and fellows. All seven of the included studies achieved a MERSQI score of at least 11.5 of 18,^19-21,28-31^ where a mean score of 10.7 is generally considered to be an acceptable cutoff value for methodological quality in medical education research.^32^ This is consistent with a systematic review by Mao et al^9^ on VR for surgical training as a whole, which showed similar findings, with a mean MERSQI score of 11.7 of 18 (1.9).

### Limitations

There are some limitations to this study that warrant mention. First, the design of the studies included in this analysis compared the performance of VR-trained and non–VR-trained groups based exclusively on cadaver-based tasks, not on live patients (Table 3). However, there is currently a paucity of literature showing improvement in real-world operating room performance after VR training.^39^

Notably, there was a limitation in the lack of a standard objective measurement tool for surgical competency because only three studies used OSATS: one study used procedure-based assessment, and others used internally developed tools and assessors to measure surgical skill and competency. Owing to the lack of a standardized measuring tool, it is difficult to assess advantages of VR training over standard training methods for various procedures that value certain factors over others. Given the high heterogeneity of procedures observed in this study, lack of standardized outcome measures made analysis of pooled data difficult. Furthermore, most of the included studies represented fewer than 25 participants and comprised mostly single-center studies, although this was largely because of the small volume of trainees of most orthopaedic surgery residency programs. Another factor limiting the conclusions we can make from this study is that only one included study by Logishetty et al^31^ assessed longitudinal and repeated application of VR modality training as opposed to isolated single-opportunity sessions with VR. This study also assessed trainee performance before and after 6 weeks VR training,^31^ with no control group and as such was excluded from pooled data analysis.

### Future Directions

Despite the promising potential of VR in the education of orthopaedic surgery trainees within arthroplasty, definitive data regarding its efficacy will be lacking without several key factors. Namely, a standardized measurement tool and application of a longitudinal VR curriculum, which shows improved performance on direct patient care and outcomes within the OR.

## Conclusion

Although the extent to which VR can outright replace traditional learning modalities is unclear at this time, its usefulness as a supplemental learning modality in arthroplasty, especially in the absence of available on-demand resources may be of value. A paucity of literature exists to evaluate the effect of a longitudinal VR curriculum on direct patient care and performance by orthopaedic surgery residents learning techniques in arthroplasty, necessitating additional study.

## Supplementary Material
