# Supplementary material for: Virtual Reality Is an Effective Tool for Learning Techniques in Arthroplasty: A Systematic Review and Meta-Analysis
Source: J Am Acad Orthop Surg Glob Res Rev. 2023 Jun 19;7(6):e23.00078. doi: 10.5435/JAAOSGlobal-D-23-00078 (PMC10284329; doi:10.5435/JAAOSGlobal-D-23-00078)
Supplement: Supplementary file 1 [file jagrr-7-e23.00078-s001.docx]

| Author & Year | Procedure | Performance Measures | Intervention | Score | | P value |
| --- | --- | --- | --- | --- | --- | --- |
| Logishetty 2020 | AA-THR | Procedural Duration | Post-VR | 36 mins (6) | | <0.001 |
|  |  |  | Pre-VR | 50 mins (9) | |  |
|  |  | Errors | Post-VR | 11 (5) | | <0.001 |
|  |  |  | Pre-VR | 48 (12) | |  |
| Zaid 2022 | UKA | OSATS (cumulative) | VR | 14.2 | | 0.59 |
|  |  |  | Control | 15.7 | |  |
|  |  | Procedure Duration | VR | 43 mins (14.3) | | 0.9 |
|  |  |  | Control | 42.4 mins (10.3) | |  |
| Logishetty 2019 | AA-THA | PBA | VR | median level 3b | | <0.001 |
|  |  |  | Control | median Level 2a | |  |
|  |  | Procedure Duration | VR | 42 mins (7) | | 0.03 |
|  |  |  | Control | 51 mins (9) | |  |
|  |  | Task specific checklist | VR | 23 (3) | | <0.001 |
|  |  |  | Control | 9 (2) | |  |
| Hooper 2019 | THA | Novel Cadaver sessions assessment | VR  (pre/post) | 79.2 (15.9) | 90.4 (27.6) | 0.386 |
|  |  |  | Control  (pre/post) | 74.7 (12.9) | 93.3 (23.4) | 0.099 |
| McKinney 2022 | UKA | Procedure Duration | VR | 26.7 (5) | | <0.01 |
|  |  |  | Control | 35.4 mins (4) | |  |
|  |  | Procedure Specific Checklist | VR | 33/36 (1.4) | | <0.01 |
|  |  |  | Control | 27/36 (3.3) | |  |
|  |  | Global Assessment 5-point Rating Scale: Time and Motion | VR | 2.36 (0.92) | | <0.01 |
|  |  |  | Control | 2.64 (0.67) | |  |
|  |  | GA: Instrument Handling | VR | 2.82 (1.08) | | 0.05 |
|  |  |  | Control | 3.73 (0.90) | |  |
|  |  | GA: Knowledge of Instruments | VR | 2.45 (1.13) | | <0.01 |
|  |  |  | Control | 3.82 | |  |
|  |  | GA: Flow of Operation and forward planning | VR | 2.36 (1.12) | | 0.01 |
|  |  |  | Control | 3.45 (0.52) | |  |
|  |  | GA: Knowledge of Specific procedure | VR | 3.00 (1.00) | | 0.12 |
|  |  |  | Control | 3.64 (0.81) | |  |
| Lohre 2020 | Reverse Shoulder | OSATS (cumulative) | VR | 15.9 (2.5) | | <0.001 |
|  |  |  | Control | 9.4 (3.2) | |  |
|  |  | Procedural Duration | VR | 17.1 mins (5.7) | | <0.01 |
|  |  |  | Control | 25.3 mins (32.5) | |  |
|  |  | Errors | VR | 0.15 | | <0.001 |
|  |  |  | Control | 0.65 | |  |
| Lohre 2020 | Glenoid Exposure | OSATS (cumulative) | VR | 11.8 (2.5) | | 0.7 |
|  |  |  | Control | 12.5 (4.8) | |  |
|  |  | Procedural Duration | VR | 14 mins (7) | | 0.04 |
|  |  |  | Control | 21 mins (6) | |  |
|  |  | Instrument handling score | VR | 3.25 (0.7) | | 0.03 |
|  |  |  | Control | 3.0 (1.8) | |  |
